# PD-1 and LAG-3-positive T cells are associated with clinical outcomes of relapsed/refractory multiple myeloma patients

**DOI:** 10.1186/s40001-022-00923-5

**Published:** 2022-12-19

**Authors:** Ming Chen, Jinlian Zhu, Xuedong Yang, Jianxin Yao, Yuqing Liu, Qiang Liu

**Affiliations:** 1 Department of Hematology, Changshu No.2 People’s Hospital, No.18, Taishan Road, Changshu, 21550 Jiangsu Province China; 2 Department of Oncology, Changshu No.2 People’s Hospital, No.18, Taishan Road, Changshu, 21550 Jiangsu Province China; 3 Department of Hematology, Changshu No.1 People’s Hospital, No.1 Shuyuan Road, Changshu, 21550 Jiangsu Province China

**Keywords:** Relapsed/refractory multiple myeloma, T cell subtype, PD-1, LAG-3

## Abstract

**Objective:**

To investigate the frequency of PD-1 and LAG-3-positive T cells in relapsed/refractory multiple myeloma (RRMM) patients and its clinical significance.

**Methods:**

This prospective observational study enrolled a total of 71 RRMM patients, as well as 70 MM patients (non-refractory) and 70 healthy individuals during January 2018 to March 2021. The frequency of circulating CD4^+^ and CD8^+^ T cells expressing PD-1 and LAG-3 was analyzed using flow cytometry. Serum cytokines of IL-6, IL-17, CRP, TNF-α and TGF-β were evaluated by enzyme linked immunosorbent assay (ELISA).

**Results:**

Significant higher 1-year mortality rate was found in RRMM patients compared with the MM patients. In both CD4^+^ and CD8^+^ T cells, the frequencies of PD-1^+^, LAG-3^+^ and PD-1^+^/LAG-3^+^ T cells were markedly higher in the RRMM patients and the deceased patients, compared with the MM patients and the survival patients, respectively. All cytokines were remarkably higher in RRMM and MM patients than in the healthy control, while only serum levels of IL-6 and IL-17 were markedly higher in RRMM patients compared with the MM patients. Positive correlation was observed among the IL-6, IL-17 and the frequencies of circulating T cells in both CD4^+^ and CD8^+^ T cells in RRMM and MM patients. The frequency of CD8^+^PD-1^+^LAG-3^+^ T cells showed the best sensitivity 82.61% and specificity 76.06% for diagnosis of RRMM using ROC curve. Meanwhile, the frequency of CD4^+^PD-1^+^ cells showed the best sensitivity 84.00% and specificity 97.35% for prediction of patients’ mortality by ROC curve. The frequencies of CD4^+^PD-1^+^, CD8^+^PD-1^+^/LAG-3^+^, as well as IL-6, IL-17 and TNF-α were found as risk factors for incidence of RRMM in all MM patients.

**Conclusion:**

The frequency of PD-1 and LAG-3-positive T cells is associated with the clinical severity and inflammation in RRMM patients, which may also serve as potential biomarkers for its diagnosis.

**Supplementary Information:**

The online version contains supplementary material available at 10.1186/s40001-022-00923-5.

## Introduction

According to the GLOBOCAN 2020 report, multiple myeloma (MM) accounts for 176,404 new cases, 9% of all cancers, and leads to 117,077 (1.2% in all cancer) cancer related death in 2020 [1]. As a plasma cell malignancy, MM remains incurable, with 5-year survival about 50% according to different studies [2, 3]. Despite the development of therapeutic strategies such as chemotherapy, autologous stem cell transplantation, targeted therapy and immunotherapy [4, 5], the treatment of relapsed/refractory multiple myeloma (RRMM) remains a clinical challenge [6, 7]. Thus, to further explore new potential targets for RRMM is still an urgent task.

In recent years, immunotherapy has been widely used in treatment of MM, including in treatment with RRMM [8, 9]. Generally, many biomarkers and molecules with negative regulation of immune function are considered to be associated with cancer-induced abnormal immune function [10]. Among these factors, program death-1 (PD-1), T-cell immunoglobulin and mucin domain-containing molecule 3 (Tim-3), and lymphocyte activation gene-3 (LAG-3) have been reported to be associated with clinical outcomes and prognosis of many cancers [11–13]. Alrasheed et al*.* found that MM patients with higher frequency of Tregs also had increased PD-1, LAG-3 [14]. However, up to now, the role of PD-1 and LAG-3, especially for LAG-3 in RRMM patients, and their relationship with patients’ inflammation response and prognosis, as well as their potential application of diagnosis for RRMM, is still unclear.

The present study aimed to investigate the frequency of PD-1 and LAG-3-positive T cells in RRMM patients and its clinical significance. This research might provide more clinical evidence for PD-1 and LAG-3 in RRMM.

## Methods and materials

### Patients

This prospective observational study enrolled a total of 71 RRMM patients who came to Changshu Second People's Hospital and Changshu First People's Hospital during January 2018 to March 2021. The diagnosis of MM was according to the guidelines for the diagnosis and management of multiple myeloma in China (2020 revision) and the criteria of National Comprehensive Cancer Network (NCCN) [15, 16]. The RRMM was defined as patients with recurrence of MM after recovery, as well as patients with  ≥ 2 standard therapies resulting in no complete recovery. The patients who were diagnosed as MM and RRMM as described above were included. The exclusion criteria were: (1) patients with other cancers; (2) patients with severe systematic diseases such as severe heart, liver or renal dysfunctions; (3) patients who received treatment of chimeric antigen receptor T cells or autologous hematopoietic stem cell transplantation before the study. Additionally, 70 patients with first diagnosed MM (non-refractory) were included as the control with the same diagnostic criteria and exclusion criteria to the above. All patients signed the written informed consent. Besides, peripheral blood samples of 70 healthy individuals were also obtained from individuals who came for physical examination. The study observed the Helsinki Declaration. Study approval was obtained from the ethical committee of Changshu Second People's Hospital and Changshu First People's Hospital.

### *Measurement of PD-1*^+^*and LAG-3*^+^*T cells*

Briefly, peripheral elbow venous blood (5 ml) was obtained from all patients within 24 h after admission. The samples were collected in tubes with heparin and added with 5 ml phosphate buffer (PBS) and then centrifuged at 1500 g for 10 min. The cell layer was then collected, added with 5 ml PBS, following with centrifugation at 1200 g for 5 min. After removing the supernatant and washing with PBS, the samples were further centrifuged at 1000 g for 2 min to obtain the peripheral blood mononuclear cells. The cells with density of 1 × 10^6^/ml were maintained in RPMI-1640 Medium (Sigma-Aldrich, St. Louis, MO, USA).

For measurement of PD-1^+^ and LAG-3^+^ T cells, flow cytometry was performed as described elsewhere [17, 18]. Antibodies used in this study included anti-CD4, anti-CD8, anti-PD-1 and anti-LAG-3 (all purchased from Abcam, USA). The measurement was conducted on a FACS Calibur flow cytometry analyzer (BD Biosciences) using Diva software (version 6.1, BD Pharmingen).

### Measurement of serum inflammatory factors

Serum inflammatory factors of IL-6, IL-17 and TGF-β were evaluated by enzyme linked immunosorbent assay (ELISA). The blood samples were collected in anticoagulant-free tubes and serum samples were obtained after centrifugation at 1500 g for 10 min. Serum levels of IL-6, IL-17, CRP, TNF-*α* and TGF-β were evaluated using commercially available kits (purchased from Abcam for IL-6, IL-17, CRP, TNF-α, and from BOSTER for TGF-β) according to the manufacturer’s instruction.

### Evaluation of other clinical outcomes and prognosis

Patients’ clinical characteristics including age, sex, body mass index (BMI), disease course, International Staging System (ISS) stage, Ig type, complications and mediation history were recorded. The study did not intervene in the treatment of any patient. All MM or RRMM patients received routine treatment according to patients’ conditions. All patients were followed up for 1 year and the survival condition was recorded.

### Statistical analysis

Data distribution was measured by Kolmogorov–Smirnov analysis. Normally and non-normally distributed data were expressed as mean ± SD or median (range), respectively. Comparison between two groups was conducted by Student’s *t* test and Mann–Whitney *U* test for normally and non-normally distributed data, respectively. Rates were analyzed by Chi-squared test. The correlation among T cells and inflammatory factors was analyzed by Spearman rank correlation analysis. ROC curve was used for diagnostic analysis. Logistic regression was conducted for risk factors of RRMM. *P * < 0.05 was regarded as statistically different. All data were calculated using SPSS 18.0.

## Results

### Basic characteristics of all patients

As shown in Table 1, the 1-year mortality rate was markedly higher in RRMM patients compared with the MM patients and the healthy control (*p* < 0.05). However, no significant difference was found for age, sex, BMI, ISS stage, disease course, and Ig type between RRMM and MM patients.

**Table 1:** Basic characteristics of all participants

Variables	RRMM (*n* = 71)	MM (*n* = 70)	Healthy (*n* = 70)	*p*_*1*_	*p*_*2*_
Age (year)	63 (48–76)	64.5 (49–76)	61.5 (48–75)	0.554	0.292
Sex (male, %)	48 (67.61)	43 (61.43)	45 (64.29)	0.361	0.620
BMI (kg/m^2^)	21.87 ± 3.70	22.32 ± 3.29	21.98 ± 3.42	0.436	0.847
ISS stage (*n*, %)				0.750	
I	14 (19.72)	16 (22.86)	–		
II	24 (33.80)	25 (35.71)			
III	33 (46.48)	29 (41.43)			
Disease course (mon)	7 (5–9)	–	–		
Ig type (n, %)				0.511	
IgG	41 (57.75)	34 (48.57)	–		
IgA	11 (15.49)	16 (22.86)			
IgM	0 (0)	0 (0)			
Light chain λ	10 (14.08)	10 (14.29)			
Light chain κ	9 (12.68)	10 (14.29)			
Mediations used before (*n*, %)					
Lenalidomide	35 (49.30)	–	–		
Dexamethasone	59 (83.10)				
Bortezomib	40 (56.34)				
Adriamycin	12 (16.90)				
1-year mortality (*n*, %)	12 (16.90)	4 (5.71)	–	0.012	–

*p*_*1*_ comparison between MM and RRMM patients; *p*_*2*_ comparison between RRMM and healthy control

Normally and non-normally distributed data were expressed as mean ± SD or median (range), respectively. Comparison between two groups was conducted by Student’s *t* test and Mann–Whitney *U* test for normally and non-normally distributed data, respectively

### Frequency of circulating T cells with different subtypes in different patients

**Fig. 1:**
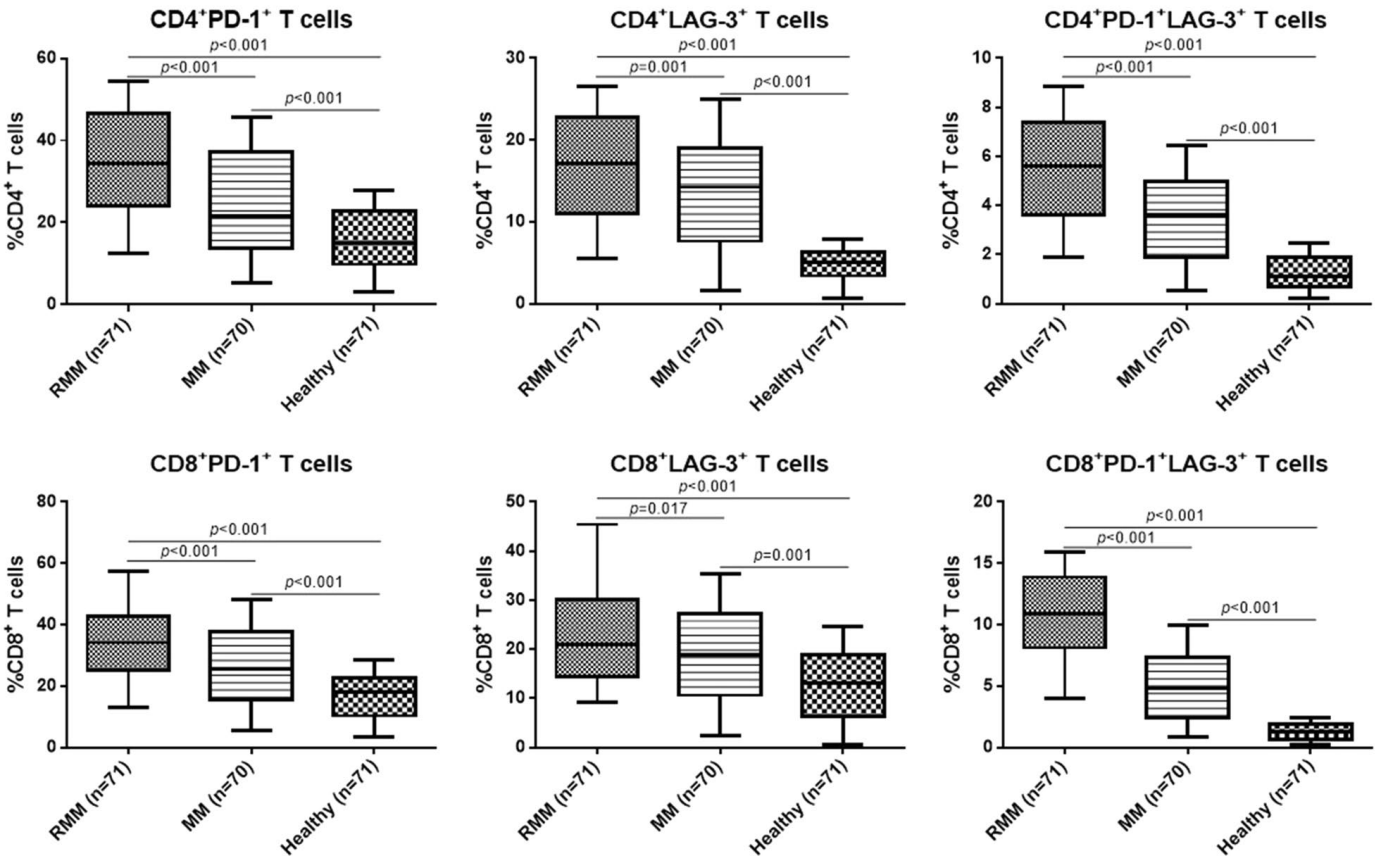
Frequency of circulating T cells with different subtypes in MM, RRMM and healthy control

**Fig. 2:**
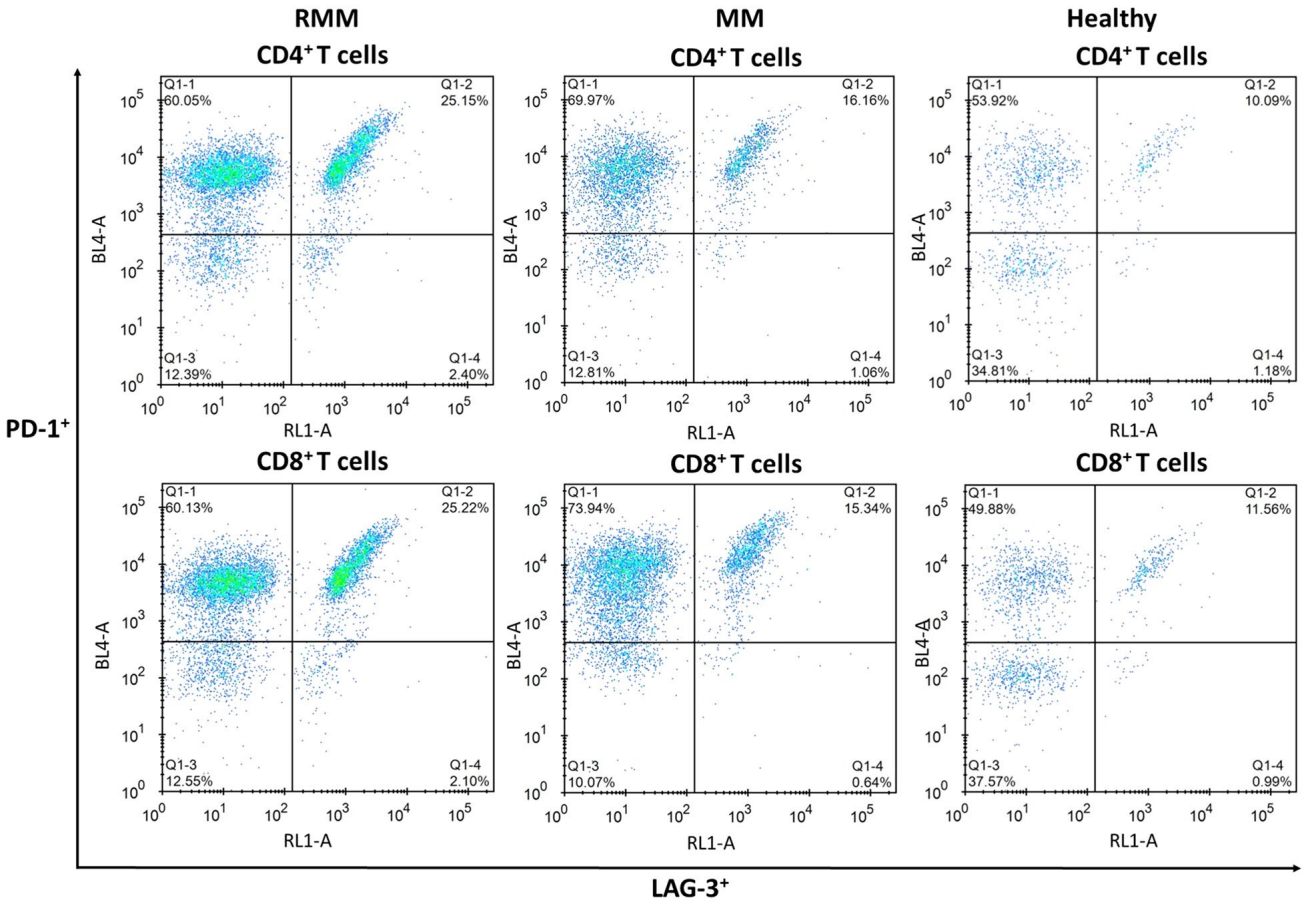
The typical flow cytometry plots of PD-1^+^/LAG-3^+^ T cells

**Fig. 3:**
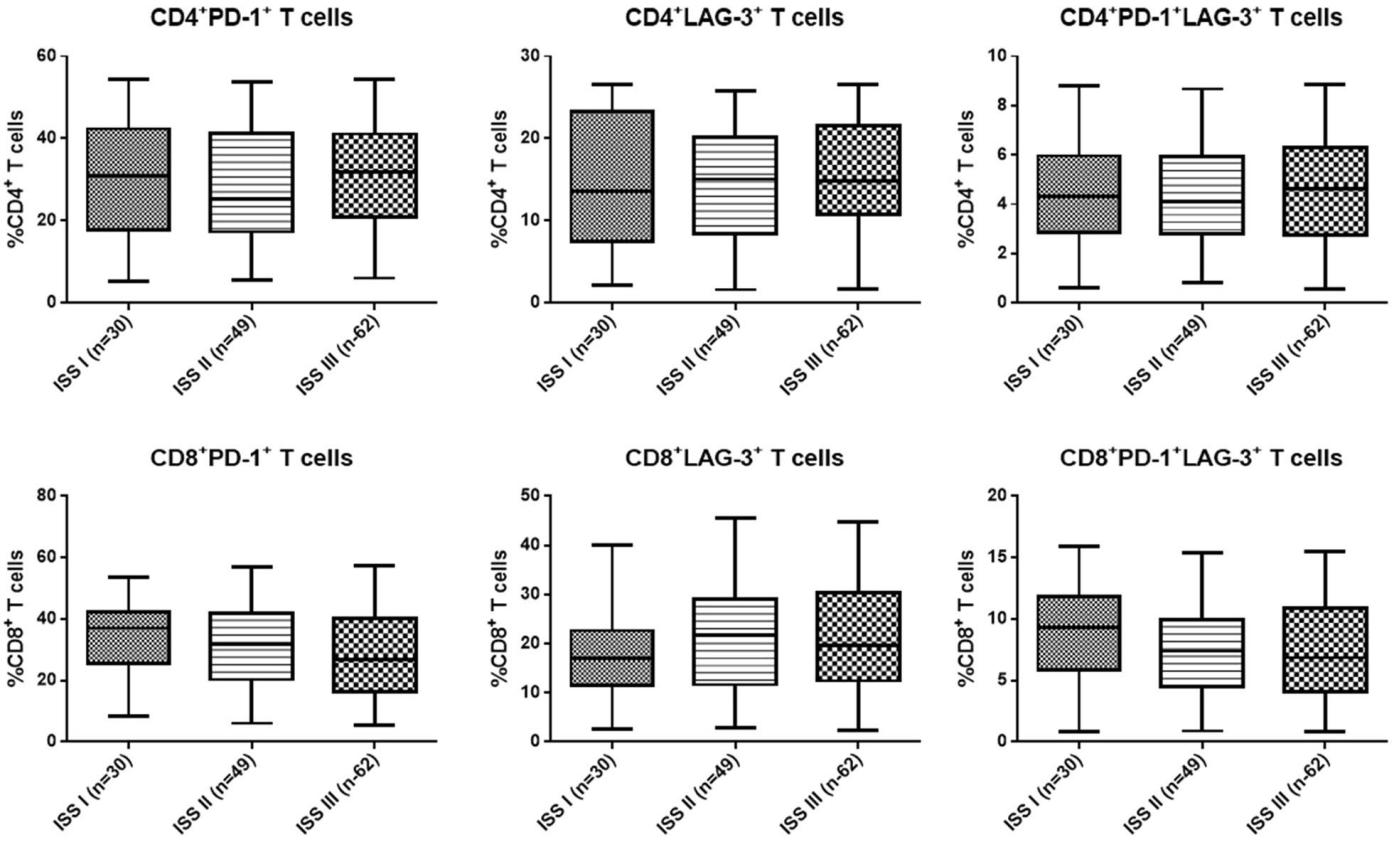
Frequency of circulating T cells with different subtypes in MM patients with different ISS stage

**Fig. 4:**
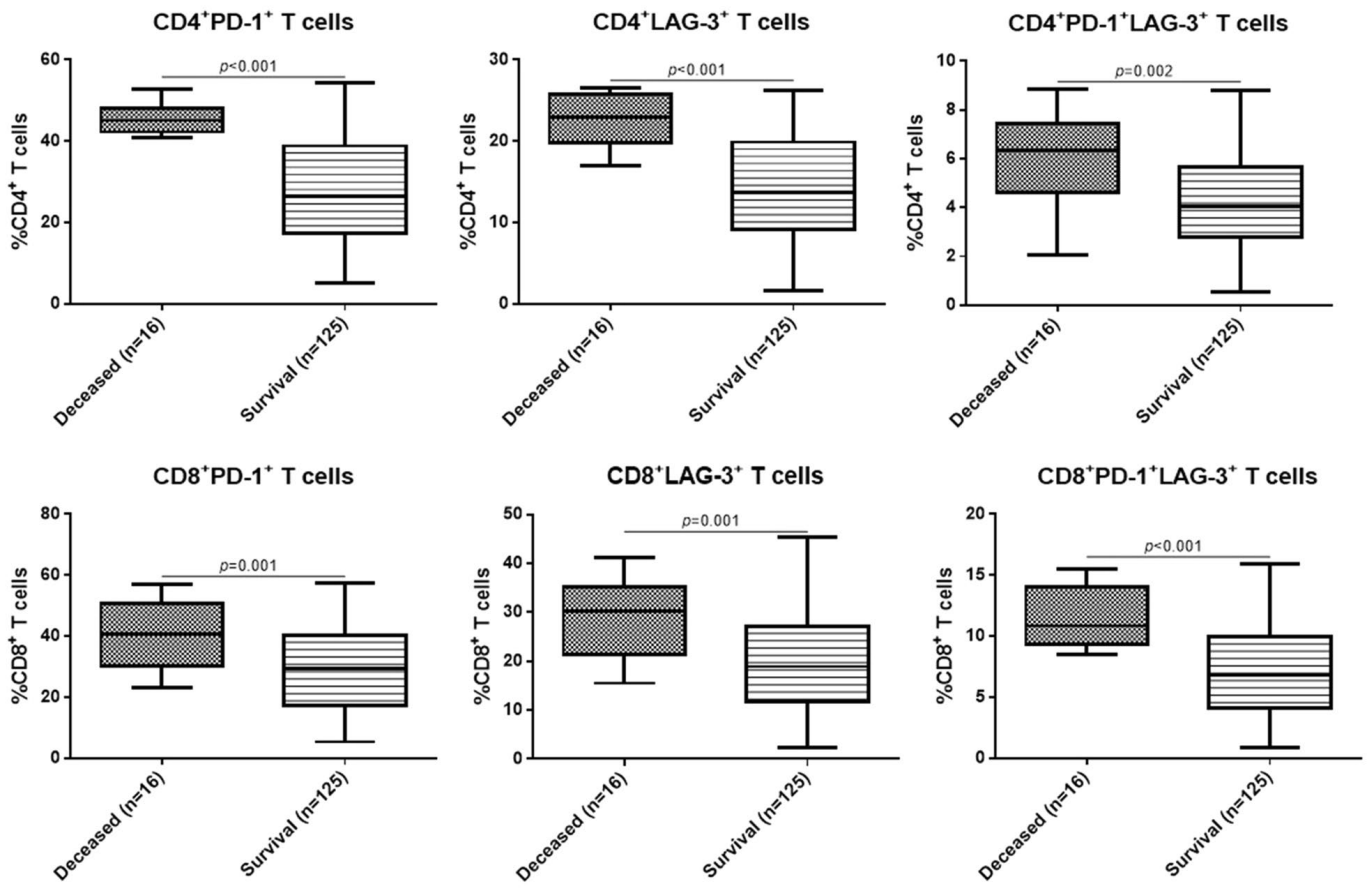
Frequency of circulating T cells with different subtypes in survival or diseased MM patients

The frequency of circulating CD4^+^ and CD8^+^ T cells expressing PD-1 and LAG-3 was then analyzed in different patients. It was found that in both CD4^+^ and CD8^+^ T cells, the frequencies of PD-1^+^ and LAG-3^+^ T cells were all markedly higher in RRMM compared with the MM patients and the healthy control (*p* < 0.05, Fig. 1). The typical flow cytometry plots of PD-1^+^/LAG-3^+^ T cells are shown in Fig. 2 with the original plots in supplementary data (Additional file 1: Fig. S1). Besides, the frequencies of PD-1^+^/LAG-3^+^ T cells were also remarkably higher in both CD4^+^ and CD8^+^ T cells of RRMM patients compared with the MM patients and healthy individuals (*p* < 0.05). Further analysis showed, in RRMM patients, frequencies of PD-1^+^ and LAG-3^+^ T, as well as PD-1^+^/LAG-3^+^ T cells in both CD4^+^ and CD8^+^ T cells showed no significant difference in ISS stage III patients compared with the patients with ISS stage I or II (*p* < 0.05, Fig. 3). However, deceased patients showed higher frequencies of the above T cell subtypes in both CD4^+^ and CD8^+^ T cells than the survival patients (*p* < 0.05, Fig. 4).

### Frequency of circulating T cells was correlated with inflammatory factors

**Fig. 5:**
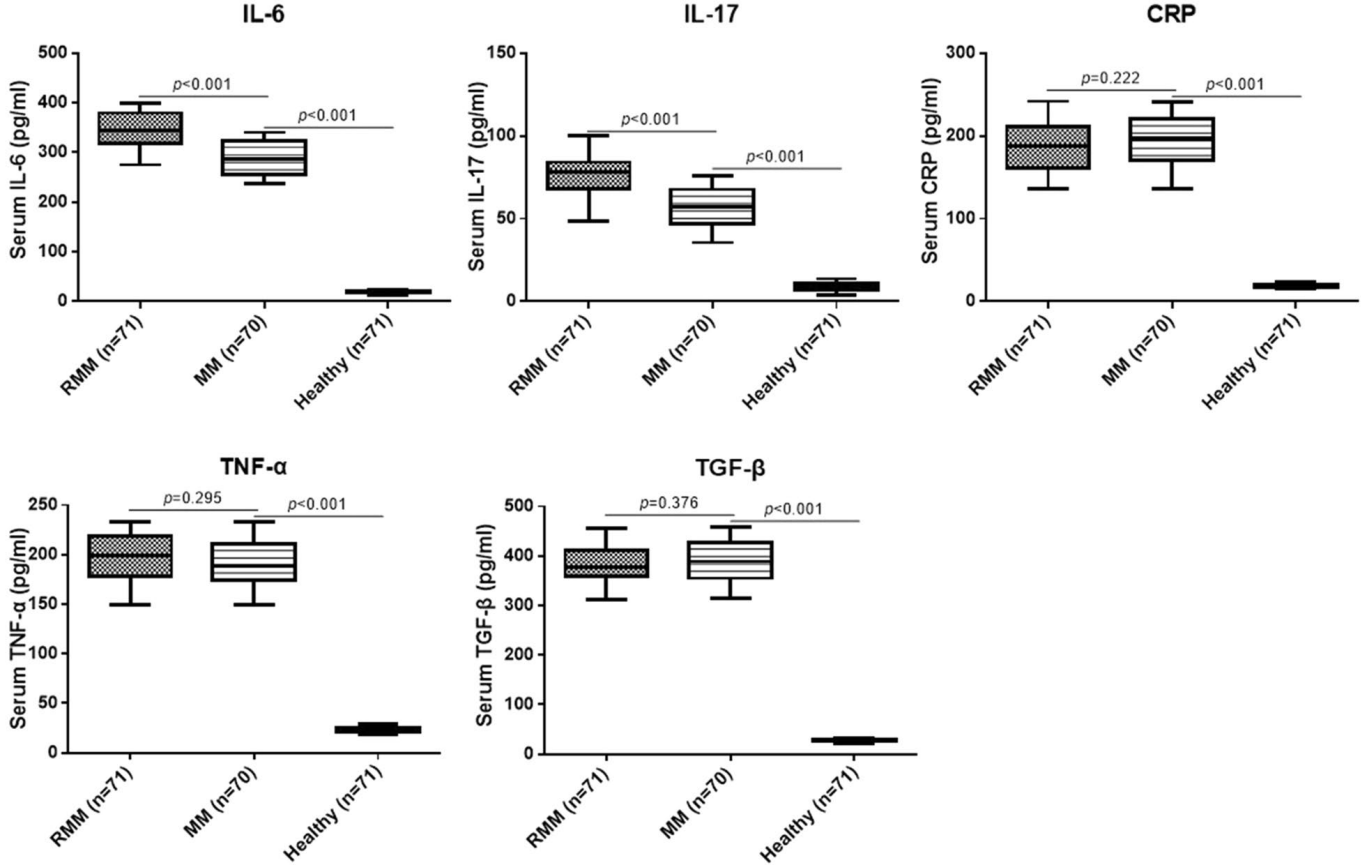
Serum levels of IL-6, IL-17, CRP, TNF-α and TGF-β in different patients

As shown in Fig. 5, it was found all inflammatory factors were remarkably higher in RRMM and MM patients than in the healthy control, while only serum levels of IL-6 and IL-17 were markedly higher in RRMM patients compared with the MM patients (*p* < 0.05). No significant difference was found for CRP, TNF-α and TGF-β between RRMM and MM patients. To further analyze the clinical significance of frequency of circulating T cells in RRMM and MM patients, Spearman’s correlation analysis was conducted between circulating T cells and inflammatory factors. Positive correlation was only observed among the IL-6, IL-17 and frequency of part of the circulating T cells in CD4^+^ and CD8^+^ T cells (Table 2).

**Table 2:** Spearman’s analysis between frequency of circulating T cells and inflammatory factors in RRMM and MM patients

		IL-6		IL-17		TGF-β		TNF-α		CRP	
		Spearman correlation	*p*	Spearman correlation	*p*	Spearman correlation	*p*	Spearman correlation	*p*	Spearman correlation	*p*
CD4^+^	PD-1^+^	0.235	0.005	0.289	0.001	− 0.068	0.421	− 0.121	0.153	0.041	0.630
	LAG-3^+^	0.164	0.052	0.142	0.093	− 0.012	0.888	0.005	0.954	− 0.032	0.702
	PD-1^+^/LAG-3^+^	0.295	< 0.001	0.242	0.004	0.046	0.591	0.199	0.018	− 0.140	0.098
CD8^+^	PD-1^+^	0.221	0.008	0.129	0.128	0.004	0.966	0.044	0.603	− 0.022	0.796
	LAG-3^+^	0.170	0.044	0.002	0.983	0.095	0.264	0.047	0.577	− 0.084	0.319
	PD-1^+^/LAG-3^+^	0.463	< 0.001	0.439	< 0.001	− 0.058	0.495	0.098	0.250	− 0.147	0.081

### Circulating T cell subtypes and diagnosis of RRMM, as well as prediction of patients’ mortality

**Fig. 6:**
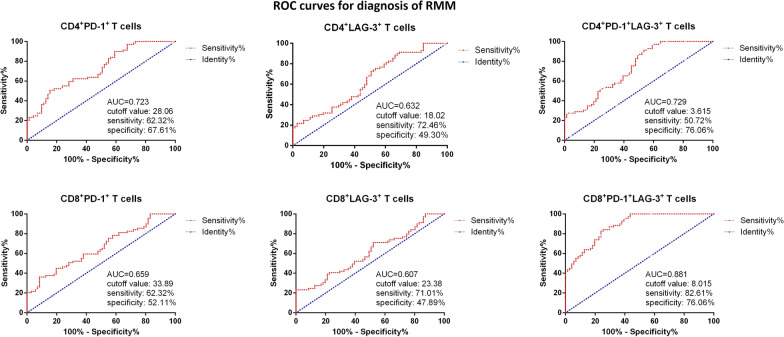
ROC for measurement of different circulating T cell subtypes for diagnosis RRMM

**Fig. 7:**
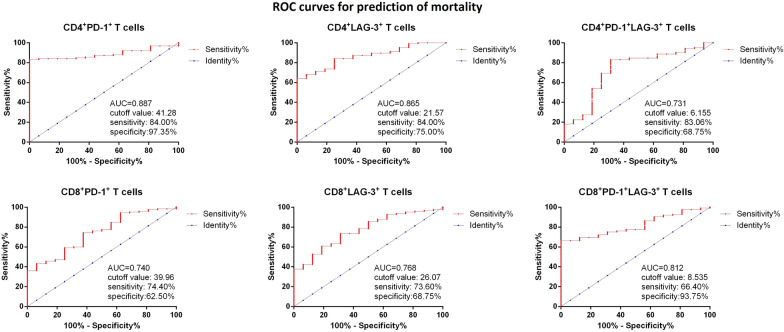
ROC for measurement of different circulating T cell subtypes for prediction of mortality

Then ROC curves were used for measurement of the sensitivity and specificity of different circulating T cell subtypes for RRMM. It was found that among the T cell subtypes, the frequency of CD8^+^PD-1^+^LAG-3^+^ T cells showed the best sensitivity 82.61% and specificity 76.06% with AUC 0.881, cutoff value 8.015% (Fig. 6). Then we also analyzed the predictive value of T cell subtypes for patients’ mortality. As shown in Fig. 7, it was found the frequency of CD4^+^PD-1^+^ cells showed the best sensitivity 84.00% and specificity 97.35% with AUC 0.887, cutoff value 41.82%.

### Circulating T cell subtype as a risk factor for RRMM

Finally, the risk factors for RRMM were analyzed using binary logistic regression by a backstep method. As shown in Table 3, it was found frequencies of CD4^+^PD-1^+^, CD8^+^PD-1^+^/LAG-3^+^, as well as IL-6, IL-17 and TNF-α were all risk factors for incidence of RRMM in all MM patients.

**Table 3:** Logistic regression for risk factors of RRMM

Variables	Wald	Odds ratio	95% CI	*p*
Age	0.592	1.016	0.975–1.059	0.442
Sex	0.620	1.327	0.656–2.684	0.431
BMI	0.668	1.041	0.945–1.147	0.414
ISS stage	0.332	0.880	0.569–1.360	0.565
Ig type	0.475	1.097	0.843–1.428	0.491
CD4^+^PD-1^+^	14.904	0.917	0.877–0.958	< 0.001
CD4^+^LAG-3^+^	1.400	0.954	0.883–1.031	0.237
CD4^+^PD-1^+^/LAG-3^+^	2.989	0.755	0.581–1.035	0.084
CD8^+^PD-1^+^	0.653	0.982	0.939–1.026	0.419
CD8^+^LAG-3^+^	< 0.001	1.000	0.943–1.060	0.997
CD8^+^PD-1^+^/LAG-3^+^	26.666	0.575	0.466–0.709	< 0.001
IL-6	19.145	0.919	0.885–0.954	< 0.001
IL-17	20.681	0.776	0.696–0.866	< 0.001
CRP	0.172	0.996	0.978–1.015	0.678
TNF-α	4.699	0.960	0.924–0.996	0.030
TGF-β	2.383	1.021	0.994–1.048	0.123

## Discussion

Despite treatment development, relapsed/refractory multiple myeloma remains a huge problem for physicians [19, 20]. Both PD-1 and LAG-3 are related to tumor immune escape. However, their relationship with RRMM and their clinical significance are not fully illustrated. In this study, we demonstrated that the higher frequency of PD-1 and LAG-3-positive T cells might be a risk factor of incidence of RRMM, and might be associated with ISS stage, prognosis and inflammation in RRMM patients, which may also serve as potential biomarkers for its diagnosis.

PD-1 is a widely used checkpoint target in cancer therapy. PD-1 checkpoint inhibitors are also used in treatment of MM, such as nivolumab and pembrolizumab [21]. Besides, PD-1-positive T cells are found to be associated with clinical characteristics of many cancers, including MM. Studies found that PD-1 and Tim-3 expressions on CD4^+^ and CD8^+^ T cells were both elevated in MM patients compared with the healthy control, especially for progressive MM patients [18]. Another study also found that before and after treatment of autologous stem cell transplant (ASCT), checkpoint molecules of PD-1, LAG-3, and Ttim-3 were all expressed on CD4^+^ and CD8^+^ T cells [17]. In a recent study, Alrasheed et al*.* demonstrated that high frequency of PD-1 + CD4 + T cells predicted higher risk for patients to develop early relapse MM [14]. In our research, we also found that the frequencies of PD-1-positive CD4^+^ and CD8^+^ T cells were markedly higher in RRMM patients, as well as deceased patients. Besides, frequency of PD-1-positive T cells were positively correlated with inflammation condition in RRMM patients, indicating that the frequency of PD-1-positive T cells was associated with both clinical outcomes and prognosis of MM patients.

Similar to PD-1, several studies also reported LAG-3 in MM. It was found that after treatment of ASCT, the mRNA levels of LAG-3 were increased in T cells of MM patients, along with increased CD4^+^LAG-3^+^ T cells, which was were associated with patients’ event-free survival [22]. Another study showed that MM patients had significantly higher levels of both PD-1 and LAG-3 than healthy control, and higher PD-1 and LAG-3 on effector T cells were correlated with shorter PFS [23]. Besides, an in vitro study by Cho et al*.* found that checkpoint molecules of PD-1, Tim-3 and LAG-3 were not persistently upregulated on CD4^+^ and CD8^+^ T cells after anti-BCMA BiTE^®^ AMG 701 treatment in MM cells [24]. However, the role of LAG-3 in RRMM is not fully illustrated. In our research, we firstly demonstrated that LAG-3-positive T cells were associated with ISS stage, 1-year mortality and inflammation in RRMM patients. Besides, the frequency of LAG-3 T cells might be a potential biomarker for incidence of RRMM. We also observed that the frequency of CD8^+^PD-1^+^LAG-3^+^ T cells has the potential for diagnosis of RMM, which implying the potential of a dual check point blockade in treatment of RMM. However, this potential needs more clinical and basic studies to confirm.

## Limitation

The study also has some limitations. First, the sample size in this study is small. Secondly, in T cell subtype analysis, we did not analyze other subtypes such as CD3^+^, CD16^+^, or naïve/memory CD4^+^ or CD8^+^ cells. Thirdly, the long-term survival analysis is still needed for relationship between different T cell subtypes and survival conditions in RRMM patients. All these need more studies to illustrate.

## Conclusion

In summary, this prospective observational study demonstrated that the frequency of PD-1^+^ and LAG-3^+^ T cells were correlated with clinical outcomes and prognosis of relapsed/refractory multiple myeloma patients, which might also be used as potential biomarkers for the relapsed/refractory stage. This study might provide new clinical evidence for the role of PD-1 and LAG-3 in multiple myeloma.

## Supplementary Information

**Additional file 1: Figure S1.** The original plots of PD-1+/LAG-3+ T cells in different patients.

## Data Availability

All data can be obtained from the authors by proper request.
