# Supplementary material for: PD-1 and LAG-3-positive T cells are associated with clinical outcomes of relapsed/refractory multiple myeloma patients
Source: Eur J Med Res. 2022 Dec 19;27:296. doi: 10.1186/s40001-022-00923-5 (PMC9761990; doi:10.1186/s40001-022-00923-5)
Supplement: Supplementary file 1 — Additional file 1: Figure S1. The original plots of PD-1+/LAG-3+ T cells in different patients. [file 40001_2022_923_MOESM1_ESM.docx]

**Supplementary data: PD-1 and LAG-3 positive T cells are associated with clinical outcomes of relapsed/refractory multiple myeloma patients**

Ming Chen^1#^ Jinlian Zhu^2#^ Xuedong Yang^1^ Jianxin Yao^1*^ Yuqing Liu^3^ Qiang Liu^1^

1.Department of Hematology, Changshu No.2 People's Hospital, Changshu, China, 215500

2.Department of Oncology, Changshu No.2 People's Hospital, Changshu, China, 215500

3.Department of Hematology, Changshu No.1 People's Hospital, Changshu, China, 215500

**#** These authors contributed equally to this work and should be considered co-first authors.

***Corresponding author:** Qiang Liu, Department of Hematology, Changshu No.2 People's Hospital, No.18, Taishan Road, Changshu City, Jiangsu P.R, China, 215500

**Email:** [csqiangliu@sina.com](mailto:csqiangliu@sina.com)

**The original plots of PD-1+/LAG-3+ T cells in different patients:**


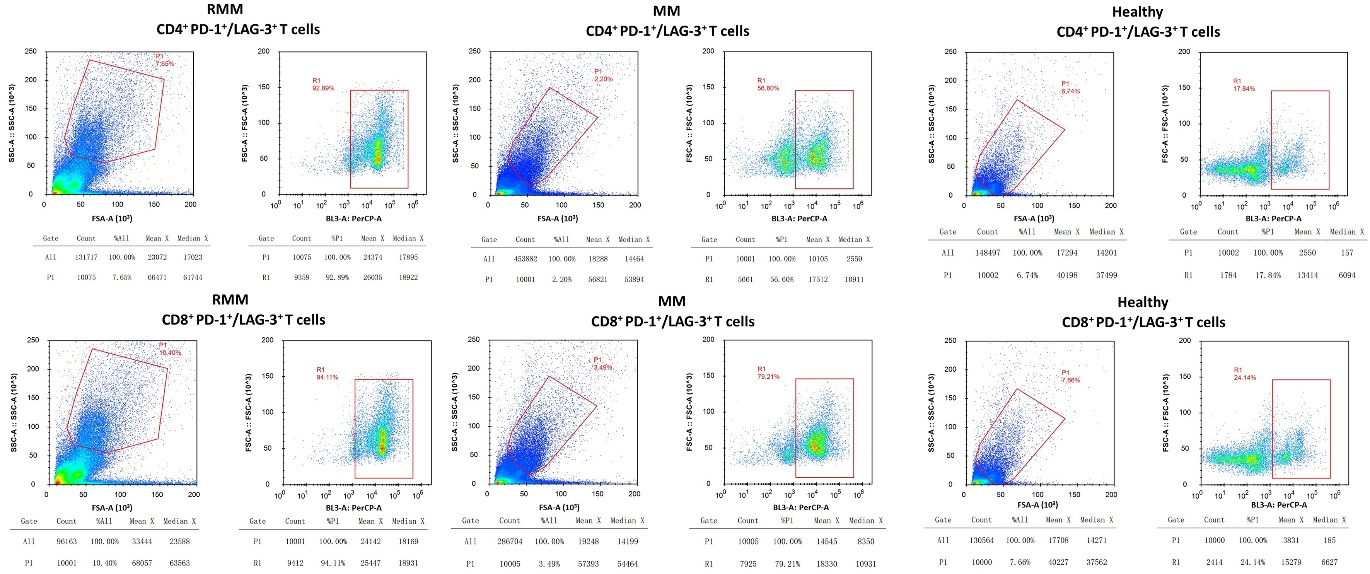


**Figure S1.** The original plots of PD-1+/LAG-3+ T cells in different patients:
